# Temporal Approach, Digitally Assisted Phacovitrectomy in a Patient with Severe Kyphosis due to Axial Spondyloarthritis

**DOI:** 10.1155/2021/5582760

**Published:** 2021-11-08

**Authors:** J. Rios-Nequis Geovanni, J. Abel Ramírez-Estudillo, L. Daniel Gutiérrez-García, Martin Jiménez-Rodríguez, Arthur Levine-Berevichez

**Affiliations:** Fundación Hospital Nuestra Señora de la Luz IAP, Mexico

## Abstract

**Purpose:**

To describe a temporal approach, digitally assisted phacovitrectomy in a patient with severe kyphosis due to axial spondyloarthritis. *Case Report*. A 70-year-old male patient with proliferative diabetic retinopathy with vitreous hemorrhage and cataract and ankylosing spondylitis with severe kyphosis. A temporal approach, digitally assisted 25 G phacovitrectomy was performed with a Constellation platform and the NGENUITY visualization system. The Trendelenburg position was utilized.

**Conclusion:**

A temporal approach, digitally assisted phacovitrectomy may be used in select cases of severe kyphosis with positive outcomes.

## 1. Introduction

Ankylosing spondylitis is part of a spectrum of axial spondyloarthritis with HLA-B27-associated autoimmune inflammatory disease that classically causes spinal involvement. As the disease progresses, there is a decrease in the mobility of the spine and thorax [1]. These patients may pose a challenge for the ocular surgical approach. Techniques that use the Trendelenburg position have been described for retinal detachment surgery with scleral buckling [2]. The positioning of the patient and the stretcher is essential to perform the procedure. Advanced cases may require up to 90° of inclination [3]. Previous reports of temporal approach cataract surgery and the Trendelenburg position have also been reported [4].

In 2010, the first 3D visualization device for retina surgery known as the Digital Assisted Vitreoretinal Surgery (DAVS) was introduced; these systems are composed of 2 cameras linked to the surgical microscope, an image processor, and a high-resolution screen. They offer the advantage of improving the ergonomics of the surgeon, increasing the depth of field, and improving the magnification [5]. We present a case report of a patient with severe kyphosis who underwent temporal approach, digitally assisted phacovitrectomy using the Trendelenburg position.

## 2. Case Report

**Figure 1:**
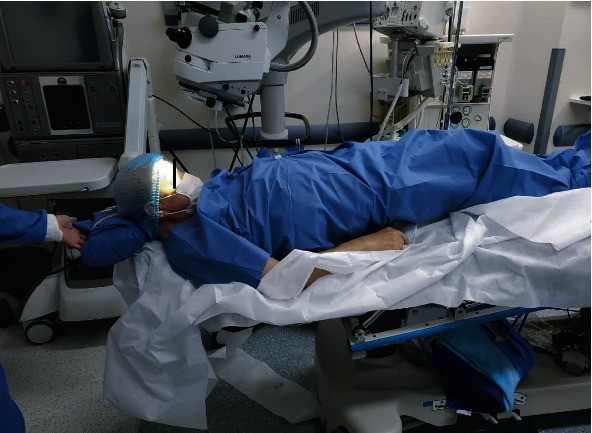
Patient in Trendelenburg position with a support pad on the head and neck in a modular surgical bed.

**Figure 2:**
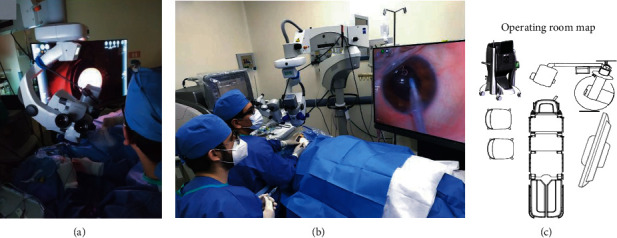
Operation room adaptation. (a) Surgical microscope OPMI Lumera 700 (Zeiss, Germany) with the RESIGHT noncontact visualization system (Zeiss, Germany) with an inclination of 30°. (b) Temporary phacovitrectomy approach with a high-resolution screen placed on one side of the surgical bed. (c) Representative map of the operating room.

A 70-year-old male patient with a medical history of type 2 diabetes mellitus, ankylosing spondylitis, and severe kyphosis was referred to our institution due to visual loss of 4 months of evolution in the right eye secondary to proliferative diabetic retinopathy (PDR), vitreous hemorrhage (VH), and cataract. The patient underwent phacovitrectomy on a modular surgical table (Operon D860, Stryker, USA); a support pad was used on the head and neck (Figure 1). The patient was positioned in the Trendelenburg position, and a temporal approach was utilized. A 25 G valved system and Constellation platform (Alcon, Fort Worth, USA) with DAVS NGENUITY visualization system (Alcon, Fort Worth, USA) were used. The OPMI Lumera 700 microscope (Zeiss, Germany) was used with the RESIGHT noncontact visualization system (Zeiss, Germany) tilted to 30° (Figure 2). The trocars were placed at M7, M9, and M11 with the phacoemulsification incision at M8 in the clear cornea. Phacoemulsification was performed through a clear corneal incision in the M8 meridian. A conventional 3-port vitrectomy was performed with previously described placement, applying panretinal photocoagulation and air tamponade without complications. The duration of the procedure was 40 minutes. The postsurgical best-corrected visual acuity was 20/40 after 12 months.

## 3. Discussion

To our knowledge, this is the first report of a temporal approach, digitally assisted phacovitrectomy using DAVS in the Trendelenburg position in a patient with severe kyphosis due to ankylosing spondylitis.

Skinner et al. reported the utility of DAVS in severe kyphosis with a superior approach. The severe kyphosis of our patient prevented us from performing a superior approach, for which we suggested doing it temporarily [6]. The DAVS system allows the high-resolution screen to be positioned at the surgeon's discretion, improving ergonomics and allowing the microscope to be adapted to inconvenient positions that could not previously be achieved with conventional microscopes. You et al. published the case of a patient with severe kyphosis with a curvature greater than 90° with rhegmatogenous retinal detachment operated with a conventional microscope; in this case, they required an extreme nonphysiological Trendelenburg position.

The Trendelenburg position allowed us a better exposure of the globe for intervention, without the negative physiological changes in cardiopulmonary function that may develop in the extreme position [7]. Close monitoring of the ventilatory and oximetric capacity is essential during the procedure. During the procedure, our patient presented adequate saturation, without presenting respiratory distress.

In conclusion, DAVS in conjunction with the Trendelenburg position may allow better patient and surgeon comfort in cases of severe kyphosis. This may allow retinal surgery to be performed more safely and efficiently in select patients.
